# A New Kinetic Modeling Approach for Predicting the Lifetime of ATH-Filled Silane Cross-Linked Polyethylene in a Nuclear Environment

**DOI:** 10.3390/polym14071492

**Published:** 2022-04-06

**Authors:** Sarah Hettal, Sébastien Roland, Konsta Sipila, Harri Joki, Xavier Colin

**Affiliations:** 1 Laboratoire Procédés et Ingénierie en Mécanique et Matériaux, Arts et Métiers Institute of Technology, CNRS, CNAM, HESAM University, 151 Boulevard de l’Hôpital, 75013 Paris, France; sarah.hettal@ensam.eu (S.H.); sebastien.roland@ensam.eu (S.R.); 2 VTT Technical Research Centre of Finland Ltd., Tekniikantie 21, Otaniemi, 02044 Espoo, Finland; konsta.sipila@vtt.fi (K.S.); harri.joki@vtt.fi (H.J.)

**Keywords:** silane cross-linked polyethylene, ATH fillers, radio-thermal oxidation, chain scissions, interfacial degradation, analytical kinetic model, lifetime prediction

## Abstract

This study focuses on the degradation of a silane cross-linked polyethylene (Si-XLPE) matrix filled with three different contents of aluminum tri-hydrate (ATH): 0, 25, and 50 phr. These three materials were subjected to radiochemical ageing at three different dose rates (8.5, 77.8, and 400 Gy·h^−1^) in air at low temperatures close to ambient (47, 47, and 21 °C, respectively). Changes due to radio-thermal ageing were investigated according to both a multi-scale and a multi-technique approach. In particular, the changes in the chemical composition, the macromolecular network structure, and the crystallinity of the Si-XLPE matrix were monitored by FTIR spectroscopy, swelling measurements in xylene, differential scanning calorimetry, and density measurements. A more pronounced degradation of the Si-XLPE matrix located in the immediate vicinity of the ATH fillers was clearly highlighted by the swelling measurements. A very fast radiolytic decomposition of the covalent bonds initially formed at the ATH/Si-XLPE interface was proposed to explain the higher concentration of chain scissions. If, as expected, the changes in the elastic properties of the three materials under study are mainly driven by the crystallinity of the Si-XLPE matrix, in contrast, the changes in their fracture properties are also significantly impacted by the degradation of the interfacial region. As an example, the lifetime was found to be approximately halved for the two composite materials compared to the unfilled Si-XLPE matrix under the harshest ageing conditions (i.e., under 400 Gy·h^−1^ at 21 °C). The radio-thermal oxidation kinetic model previously developed for the unfilled Si-XLPE matrix was extended to the two composite materials by taking into account both the diluting effect of the ATH fillers (i.e., the ATH content) and the interfacial degradation.

## 1. Introduction

Cross-linked low-density polyethylene (XLPE) is widely used in industry because of its low cost, easy processability, chemical resistance to many chemical reagents, lightness, and great flexibility [1]. This polymer is also well known to be an excellent dielectric material [2,3]. For this reason, it is commonly used as an insulating material in electrical cables. In order to improve its thermomechanical properties and reduce its production cost, several types of fillers can be incorporated into the XLPE matrix [4]. The additional advantage of aluminum tri-hydrate (ATH), whose common chemical formula is [Al_2_O_3_, 3H_2_O] or Al(OH)_3_, is that it also provides flame retardancy. Indeed, ATH fillers are well known to dissociate into aluminum trioxide (Al_2_O_3_) and water (H_2_O) according to complex endothermic processes when the temperature progressively increases from 80 °C to 1200 °C [5], with their maximum dehydration rate located at around 310–320 °C.

The main weakness of XLPE is clearly its fairly high sensitivity to oxidation [6,7,8,9,10,11]. For this reason, this polymer is generally protected by one or more antioxidants [12,13,14,15,16,17,18]. In a nuclear environment, oxidation of hydrocarbon polymers can be initiated by the radiolytic decomposition of the covalent bonds composing the repeating constitutive unit (mainly C–H bonds), but also by the thermal decomposition of hydroperoxides [19,20]. For this reason, this chemical reaction is commonly called “radio-thermal oxidation”. According to the literature, the radio-thermal oxidation of XLPE leads to a predominance of chain scissions over cross-linking, which progressively destroys the elastically active chains of the macromolecular network [8,10,11] and can cause the appearance of chemi-crystallization phenomena [10,11]. These two structural changes, i.e., chain scissions and chemi-crystallization, are directly responsible for the sudden and catastrophic embrittlement of XLPE [8,10,11]. All these degradation mechanisms have been extensively studied in the literature over the last three decades and are now fairly well understood. Kinetic models derived from these mechanisms are under development and have already started to demonstrate their excellent predictive value [11]. However, structural end-of-life criteria are still to be determined, in order to non-empirically predict the lifetime of XLPE.

In our opinion, the effect of ATH fillers on the radio-thermal oxidation of hydrocarbon polymers, and on ethylene-based polymers in particular, has been insufficiently investigated. Indeed, the scarce studies available in the literature are focused on only two types of peroxide cross-linked matrices: an ethylene propylene diene terpolymer (EPDM) composed of 71 wt.% ethylene, 28.5 wt.% propylene, and 0.5 wt.% of ethylidene norbornene [21]; and a blend of 80 wt.% of ethylene vinyl acetate (EVA) with 20 wt.% of EPDM (the latter composed of 85 wt.% ethylene, 10 wt.% propylene, and 5 wt.% ethylidene norbornene) [22,23]. In these studies, it was observed that the ATH fillers significantly accelerate the global oxidation kinetics of the polymer matrix. As an example, Colombani et al. [23] showed that the introduction of 60 wt.% of untreated ATH fillers into EVA/EPDM leads to a reduction in its oxidation induction time by about 5% and an increase in its oxidation rate by about 63% in air at 100 °C. As expected, this acceleration of the global oxidation kinetics leads to deeper changes in the macromolecular network structure, and thus to an even more premature failure of the composite materials compared to the unfilled matrix.

To further explain this early embrittlement, in these three studies it was also assumed that the oxidative degradation of the polymer matrix is faster in the immediate vicinity of the ATH fillers, thus progressively leading to ATH/polymer debonding. According to Colombani et al. [23], debonding would be favored by the poor interfacial adhesion (involving purely physical interactions) initially existing between the untreated ATH fillers and polyolefins [24]. To further explain the heterogeneity of oxidation, Planes et al. [21] assumed that γ-irradiation can cause the dehydration of ATH fillers, thus generating very reactive radical species (i.e., hydroxyl radicals HO^•^) on their surface. This additional source of radicals would lead to a higher concentration of chain scissions in the polymer matrix located in the immediate vicinity of the ATH fillers. In addition, it would be responsible for the destruction of some of the covalent bonds initially formed at the ATH/polymer interface in the case where the surface of ATH fillers had previously been treated with vinyl triethoxy silane as chemical coupling agent [21]. It should be mentioned that the same behavioral trends were observed for the radio-thermal ageing of the same silica-filled EPDM, which led the authors to reiterate these assumptions [25]. However, it was shown by Guzzo and De Paoli [26] that the ATH fillers also have a significant effect on the photo-oxidation in air at room temperature of other types of peroxide cross-linked EPDM matrices, which would imply that the interfacial mechanism is more general than announced and could be triggered by a temperature increase as well as by (solar or ionizing) irradiation.

To conclude this brief literature survey, although there is no doubt that ATH fillers significantly sensitize ethylene-based polymers to oxidation, leading to an earlier and faster alteration of their mechanical properties, the assumption of interfacial degradation still remains to be experimentally proven. In our opinion, the corresponding degradation mechanism also remains to be correctly identified. Once the interfacial degradation has been elucidated, it will then be possible to extend the kinetic models previously established for the unfilled matrix to composite materials. Of course, new end-of-life criteria, taking into account the matrix degradation both inside and outside the interfacial region, must also be proposed to predict the lifetime of the composite materials.

The objective of this research is to meet these challenges in the case of the radio-thermal ageing of an ATH-filled silane cross-linked polyethylene (Si-XLPE). The effect of three ATH contents (0, 25, and 50 phr) on the changes in the physico-chemical and mechanical properties is carefully and accurately investigated using several complementary analytical methods, in order to identify the structural changes occurring both inside and outside the interfacial region. Then, the role of the ATH fillers is introduced into the radio-thermal oxidation kinetic model previously developed for the unfilled Si-XLPE matrix [11], in order to predict the changes in elastic and fracture properties and the lifetimes of the composite materials.

## 2. Materials and Methods

### 2.1. Materials

Films of thickness about 500 μm of Si-XLPE matrix filled with three different ATH contents: 0, 25, and 50 phr, were directly provided by Nexans NRC (Lyon, France). ATH fillers were first incorporated into a linear low-density polyethylene grafted with vinyl trimethoxy silane side groups (Si-g-LDPE) using a continuous mixer. Then, the formulated mixture was molded by extrusion into films before cross-linking the polymer matrix by immersion in water at 65 °C for 48 h [27].

As soon as they were received, the composite films were characterized by several common laboratory techniques, in order to determine their ATH content more precisely but also to access other key physico-chemical properties characterizing the initial state of their Si-XLPE matrix, such as density, crystallinity ratio, and gel content. The values of all these physico-chemical properties are reported in Table 1 for the three materials under study.

The ATH fillers were not characterized in this study, but their technical data sheet contains crucial information that is worth recalling here. Their average diameter, density, and (BET) specific surface area were about 1.6 µm, 2.42 g·cm^−3^, and 4 m^2^·g^−1^, respectively. It should also be emphasized that these fillers did not contain any chemical coupling agent on their surface.

### 2.2. Radio-Thermal Ageing Conditions

Radio-thermal ageing was performed in the Panoza and Roza facilities at UJV Rez, Czech Republic, using a ^60^Co γ-ray source at different temperatures. All the exposure conditions are summarized in Table 2. It should be specified that the radio-thermal ageing experiments were performed at three distinct dose rates (8.5, 77.8, and 400 Gy·h^−1^) and low temperatures close to ambient (47 °C, 47 °C, and 21°C, respectively), in order to investigate the effect of dose rate on the global oxidation kinetics. The withdrawal times and doses at which the samples were removed from the irradiation facilities are given in the two last columns of Table 2.

### 2.3. Experimental Characterizations

#### 2.3.1. FTIR Spectroscopy

FTIR spectroscopy was used in ATR mode to monitor and titrate the oxidation products, in particular the carbonyl products, throughout radio-thermal exposure, due to the presence of ATH fillers and the rather large thickness of the films to be analyzed (about 500 µm). FTIR spectra were recorded from 4000 to 650 cm^−1^ with a PerkinElmer FTIR Frontier spectrometer (PerkinElmer, Villebon-sur-Yvette, France) equipped with a diamond/ZnSe crystal, after averaging 16 scans obtained with a resolution of 4 cm^−1^.

**Figure 1:**
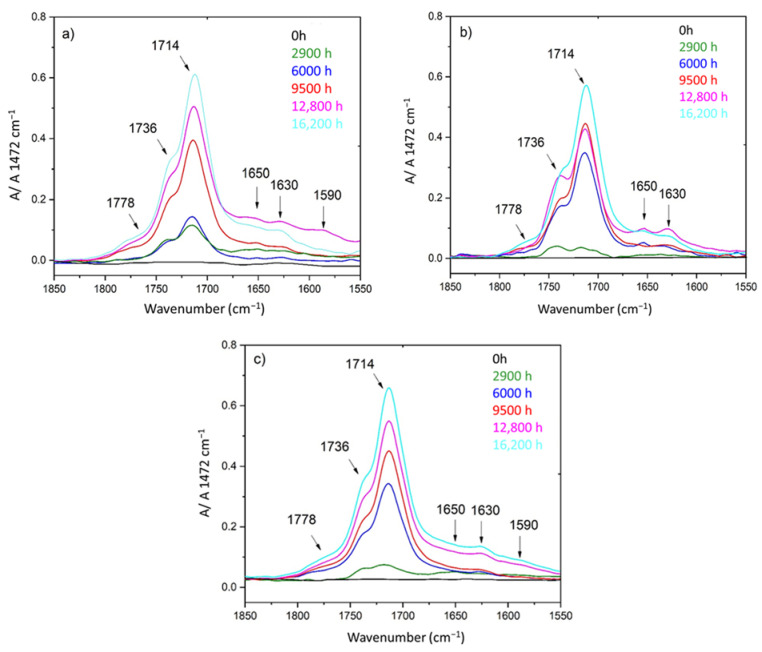
Changes in the FTIR spectrum (obtained in ATR mode) of the Si-XLPE matrix filled with 0 phr (**a**), 25 phr (**b**), and 50 phr (**c**) of ATH, during radio-thermal exposure in air under 8.5 Gy·h^−1^ at 47 °C.

As already shown for the unfilled Si-XLPE matrix [11], radio-thermal oxidation of the two composite materials leads to the formation of a wide variety of carbonyl products detected through the early appearance and rapid growth of a multi-contribution broad band, typically ranging between 1650 and 1850 cm^−1^, in the FTIR spectrum. As an example, the corresponding spectral changes occurring in air under the lowest dose rate (8.5 Gy·h^−1^) for the three ATH contents under study are reported in Figure 1. In all three cases, the fact that this broad band is centered at about 1714 cm^−1^ means that carboxylic acids are the main contributors, presumably due to their much higher coefficient of molar extinction compared to other types of carbonyl products [28,29]. However, two shoulders can clearly be observed at about 1736 and 1778 cm^−1^, indicating that many other carbonyl products, e.g., aldehydes, and linear and cyclic esters (i.e., γ-lactones) or anhydrides, are also formed as secondary contributors. Finally, the slower development of three additional IR bands can also be observed at around 1590, 1630, and 1650 cm^−1^. The first two bands were assigned to conjugated carbon–carbon double bonds, with the conjugation degree being a decreasing function of the wavenumber, whereas the last one was assigned to isolated carbon–carbon double bonds [30]. All these IR bands and their assignments are reported in Table 3.

It should be mentioned that the same spectral changes were observed for the two other dose rates (i.e., 77.8 Gy·h^−1^ and 400 Gy·h^−1^) under investigation.

Finally, the absorbance of carboxylic acids at about 1714 cm^−1^ was chosen to determine the global oxidation kinetics of the Si-XLPE in this study. It should be recalled that, in principle, FTIR in ATR mode is not a quantitative technique, because large absorbance variations can be measured for the same sample depending on many factors such as penetration depth of the IR beam, surface aspect of the sample (roughness, color, etc.), spatial distribution of ATH fillers, etc. To try to eliminate all these variations, it was decided to normalize the absorbance of carboxylic acids with the absorbance of the CH_2_ scissoring vibration at 1472 cm^−1^, assigned to the PE crystal phase [31,32] and thus assumed to be almost completely insensitive to thermal ageing [18]. This absorbance ratio is called the “carboxylic acid index” hereafter.

#### 2.3.2. Differential Scanning Calorimetry

Differential scanning calorimetry (DSC) was used for two purposes: to monitor and titrate hydroperoxides (POOH) and to highlight the changes in crystalline morphology throughout the radio-thermal exposure. DSC thermograms were recorded with a TA Instruments Q1000 DSC calorimeter (TA Instruments, Guyancourt, France) previously calibrated with an indium reference. Film samples with a mass ranging between 5 and 10 mg were introduced into a closed standard aluminum pan to be analyzed between 50 °C and 250 °C with a heating rate of 10 °C·min^−1^ under a nitrogen flow of 50 mL·min^−1^.

**Figure 2:**
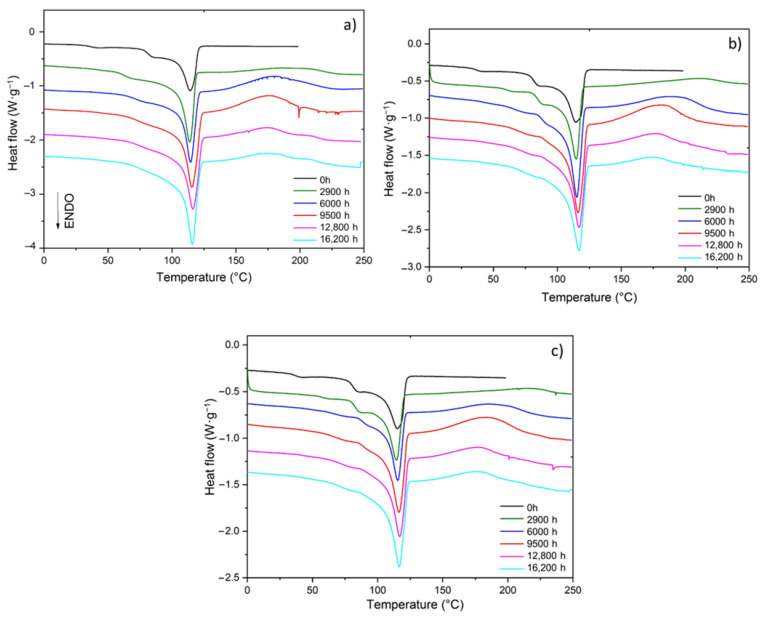
Changes in the DSC thermogram of the Si-XLPE matrix filled with 0 phr (**a**), 25 phr (**b**), and 50 phr (**c**) of ATH, during radio-thermal exposure in air under 8.5 Gy·h^−1^ at 47 °C.

As already shown for the unfilled Si-XLPE matrix [11], the radio-thermal oxidation of the two composite materials leads to the early appearance and rapid growth of an exothermic peak above the melting zone of the crystalline phase. This new peak is typically in the range between 130 °C and 230 °C and corresponds to POOH decomposition in the DSC cavity. As an example, the changes in the DSC thermogram in air under the lowest dose rate (8.5 Gy·h^−1^) for the three ATH contents under study are reported in Figure 2.

The concentration of hydroperoxides was determined as follows:

(1) $$\left[\mathrm{POOH}\right]=\frac{1}{1-X_{\mathrm{ATH}}}\;\frac{\Delta H_{\mathrm{POOH}}}{\Delta H_{\mathrm{theory}}}$$

where $\Delta H_{\mathrm{POOH}}$ is the area under the exothermic peak between 130 °C and 230 °C on the DSC thermogram, $\Delta H_{\mathrm{theory}}$ is the theoretical value of the decomposition enthalpy of POOH, which can be calculated from the classical theoretical concepts of thermochemistry or determined experimentally from POOH model compounds ($\Delta H_{\mathrm{theory}}=$ 291 kJ·mol^−^^1^ [29]), and $X_{\mathrm{ATH}}$ is the mass fraction of ATH fillers in the composite material under consideration, whose values are reported in Table 1.

It should be pointed out that the denominator ($1-X_{\mathrm{ATH}}$) was introduced into Equation (1) for calculating the POOH concentration in the 100% Si-XLPE matrix, which then allowed the results obtained for the three ATH contents under study to be compared.

As already shown for the unfilled Si-XLPE matrix [11,33], the radio-thermal oxidation of the two composite materials leads to a predominance of chain scissions over cross-linking. Chain scissions progressively destroy the elastically active chains of the macromolecular network, thus producing small, linear macromolecular fragments. As the amorphous phase of Si-XLPE is in a rubbery state, these short fragments can easily migrate towards crystalline lamellae to induce a chemi-crystallization, i.e., a lamellar thickening and an increase in crystallinity. In general, these short fragments preferentially integrate into the thinnest crystalline lamellae, which leads to a narrowing of the lamellar size distribution around a maximum thickness and thus a harmonization of the crystalline morphology. The direct consequences of these two morphological changes, i.e., lamellar thickening and an increase in crystallinity, are clearly highlighted on the DSC thermograms in Figure 2. Indeed, the progressive disappearance of the secondary melting peaks constituting the low temperature part (typically between 40 °C and 100 °C) of the wide melting range can be observed. This disappearance correlates well with the increase in the height (and thus in the area) of the main melting peak, while its melting temperature remains almost constant around its initial value (≈114.7 ± 0.6 °C). 

The crystallinity ratio $X_{C}$ was determined as follows:

(2) $$X_{C}=\frac{1}{1-X_{\mathrm{ATH}}}\;\frac{\Delta H_{m}}{\Delta H_{m0}}\times 100$$

where $\Delta H_{m}$ is the sum of the areas under the endothermic peaks observed between 40 and 125 °C on the DSC thermogram, and $\Delta H_{m0}$ is the melting enthalpy of the PE crystal. According to the literature, $\Delta H_{m0}=$ 292 J·g^−^^1^ [34].

#### 2.3.3. Density Measurements

Density measurements made throughout radio-thermal exposure provided very valuable information at two different structural scales because the changes in density of the films resulted from changes in both the chemical composition of the amorphous phase (due to oxidation) and the crystalline morphology of the Si-XLPE matrix (due to chemi-crystallization) [33]. In this study, these measurements were used to access the changes in the volume fraction of crystals V_C_ and the changes in the density of the amorphous phase ρ_a_ of the Si-XLPE matrix.

The density of the films was determined through hydrostatic weighing at room temperature (23 °C) using a Mettler Toledo MS104TS microbalance (Mettler Toledo SAS, Viroflay, France). Rectangular film samples were first weighed in air, then following immersion in ethanol, and their densities were determined by applying Archimedes’ principle:

(3) ρcomp=mAirmAir−mImρEth

where $m_{\mathrm{Air}}$ and mIm are the sample weights in air and immersed in ethanol, respectively, and $\rho_{\mathrm{Eth}}$ is the density of ethanol at 23 °C ($\rho_{\mathrm{Eth}}=$ 0.789 [35]).

The density of the Si-XLPE matrix was deduced as follows:

(4) $$\rho =\frac{\rho_{\mathrm{comp}}-V_{\mathrm{ATH}}\rho_{\mathrm{ATH}}}{1-V_{\mathrm{ATH}}}$$

where $\rho_{\mathrm{ATH}}$ is the density of ATH fillers ($\rho_{\mathrm{ATH}}=$ 2.42), and $V_{\mathrm{ATH}}$ is their volume fraction in the composite material under consideration, with values as reported in Table 1.

Then, $V_{C}$ was deduced from $X_{C}$ and ρ as follows:

(5) $$V_{C}=\frac{\rho }{\rho_{C}}X_{C}$$

where $\rho_{C}$ is the density of the PE crystal. According to the literature, $\rho_{C}=$ 1.014 [36].

Finally, $\rho_{a}$ was deduced from $V_{C}$ and ρ as follows:

(6) $$\rho_{a}=\frac{\rho -V_{C}\rho_{C}}{1-V_{C}}\;$$

For reference, in the literature, a typical value for PE is $\rho_{a}=$ 0.85 [37].

The initial values determined for $V_{C}$ and $\rho_{a}$ are reported in Table 4. These values can be considered to be similar for the three materials under study because the slight differences are of the order of magnitude of the experimental scattering. The average values retained for the three materials under study are given in the last line of Table 4.

#### 2.3.4. Swelling Measurements

Swelling measurements were performed to determine the concentration of elastically active chains in the Si-XLPE macromolecular network throughout the radio-thermal exposure and thus to deduce the corresponding concentration of chain scissions.

Film samples with an initial mass m_i_ of about 30 mg were introduced into xylene, previously heated to 130 °C, for 24 h, until their equilibrium masses were reached after complete swelling $m_{s}$. The samples were then dried under vacuum at 80 °C for 48 h, in order to determine their masses without the soluble fraction $m_{d}$. The corresponding masses $m_{\mathrm{sg}}$ and $m_{\mathrm{dg}}$ for the swollen and dried gel were deduced as follows:

(7) $$m_{\mathrm{sg}}=\frac{m_{s}}{1-X_{\mathrm{ATH}}}$$

(8) $$m_{\mathrm{dg}}=\frac{m_{d}}{1-X_{\mathrm{ATH}}}\;$$

For the unfilled Si-XLPE matrix, the common Flory–Rehner’s theory [38,39] was used to determine the concentration of elastically active chains:

(9) $$\nu =-\frac{1}{V_{\mathrm{sol}}}.\;\frac{\ln\left(1-V_{r0}\right)+V_{r0}+{\chi V}_{r0}^{2}}{V_{r0}^{\frac{1}{2}}-\frac{2V_{r0}}{f}}\;$$

where $V_{\mathrm{sol}}$ is the molar volume of xylene ($V_{\mathrm{sol}}=139.3$ cm^3^·mol^−1^), χ is the Flory–Huggins interaction parameter between xylene and Si-XLPE (χ= 0.31 [8,40]), f is the crosslink functionality (f=4 for Si-XLPE), and $V_{r0}$ is the volume fraction of polymer in the swollen Si-XLPE network, which can be expressed as:

(10) $$V_{r0}=\frac{1}{1+\frac{\left(\frac{m_{\mathrm{sg}}}{m_{\mathrm{dg}}}-1\right).\rho_{\mathrm{pol}}}{\rho_{\mathrm{sol}}}}\;$$

where $\rho_{\mathrm{pol}}$ and $\rho_{\mathrm{sol}}$ are the densities of Si-XLPE ($\rho_{\mathrm{pol}}=$ 0.806) and xylene ($\rho_{\mathrm{sol}}=$ 0.761), respectively, at 130 °C [8,40].

For the two composite materials, however, the Flory–Rehner’s theory modified by Kraus [41,42] was used to calculate ν, since we know that ATH fillers oppose, and thus limit, the swelling of the Si-XLPE matrix:

(11) $$\nu =-\frac{V_{r0}}{V_{\mathrm{sol}}}.\frac{\ln\left(1-V_{r}\right)+V_{r}+{\chi V}_{r}^{2}}{V_{r}^{\frac{1}{3}}V_{r0}^{\frac{2}{3}}-\frac{2V_{r}}{f}}$$

where $V_{r}$ is the volume fraction of polymer in the swollen ATH-filled Si-XLPE network.

Neglecting, as a first approach, an eventual chemical cross-linking process, the concentration of chain scissions throughout the radio-thermal exposure was simply deduced as follows [43]:

(12) $$S=\nu -\nu_{\mathrm{ini}}$$

where ν and $\nu_{\mathrm{ini}}$ are the concentrations of the elastically active chains after and before radio-thermal ageing, respectively.

#### 2.3.5. Micro-Indentation

The consequences of radio-thermal oxidation on the elastic properties were determined by micro-indentation. The films were cut in their thickness direction and embedded in a commercial acrylic KM-V resin, which was cross-linked for 12 h under primary vacuum at room temperature. Then, the film cross sections were polished with a MECAPOL P320 device (PRESI SA, Eybens, France) using silicon carbide abrasive papers of decreasing particle size (typically from 80 to 2400 granulometry). Finally, a mirror finish was obtained using diamond pastes of decreasing particle size (typically from 3 to 0.25 μm).

The indentations were performed on the polished cross sections using an Anton Paar Micro Hardness Indenter (Anton Paar, Les Ulis, France) equipped with a Vickers diamond tip of pyramidal geometry, with a force of 450 mN and a loading and unloading rate of 1000 μm·min^−1^. A pause of 10 s was systematically applied between loading and unloading to eliminate the viscous response of the Si-XLPE matrix. The Indentation 4.37 operating software gave the value of the reduced modulus $E_{\mathrm{red}}$ of the material directly, calculated according to Oliver and Pharr’s method [44,45,46]:

(13) $$E_{\mathrm{red}}=\frac{\sqrt{\pi }\;\frac{\Delta F}{\Delta h}}{2\beta \sqrt{A_{c}}}$$

where ΔF/Δh is the slope at the origin point of the unloading curve, β is a shape factor depending on the indenter type (β= 1.012 for a Vickers tip), and $A_{c}$ is the contact area between the indenter and the sample, projected perpendicularly to the indenter axis on the sample surface: $A_{c}=a^{2}$, where a is the side length of the projected square. This last quantity is also directly provided by the operating software. It depends on both the penetration depth of the indenter and the indenter geometry.

The local elastic modulus $E_{\mathrm{comp}}\left(j\right)$ was deduced from the reduced modulus $E_{\mathrm{red}}$ as follows:

(14) $$E_{\mathrm{comp}}\left(j\right)=\frac{1}{\frac{1-\vartheta^{2}}{E_{\mathrm{red}}}-\frac{1-\vartheta_{\mathrm{ind}}^{2}}{E_{\mathrm{ind}}}}$$

where ϑ is the Poisson’s ratio of the unaged material under consideration (ϑ= 0.42 for the unfilled Si-XLPE matrix and ϑ= 0.45 for the two composite materials), and $\vartheta_{\mathrm{ind}}$ and $E_{\mathrm{ind}}$ are the Poisson’s ratio ($\vartheta_{\mathrm{ind}}=$ 0.07) and the Young’s modulus of the diamond tip ($E_{\mathrm{ind}}=$ 1141 GPa), respectively.

Profiles of the elastic modulus were determined over the film thickness with an indentation step of 50 µm. Then, the average elastic modulus of each film $E_{\mathrm{comp}}$ was deduced by averaging the N local values $E_{\mathrm{comp}}\left(j\right)$ constituting the micro-indentation profile:

(15) $$E_{\mathrm{comp}}=\frac{1}{N}\overset{N}{\underset{j=1}{\sum }}E_{\mathrm{comp}}\left(j\right)$$

As shown in previous studies [45,46], $E_{\mathrm{comp}}$ can roughly be associated with the Young’s modulus of the composite material under consideration.

Finally, the elastic modulus of the Si-XLPE matrix was deduced using the common Guth–Gold’s equation [47]:

(16) $$E=\frac{E_{\mathrm{comp}}}{1+2.5\times V_{\mathrm{ATH}}+14.1\times V_{\mathrm{ATH}}^{2}}$$

#### 2.3.6. Uniaxial Tensile Testing

The consequences of radio-thermal oxidation for the fracture properties were determined by uniaxial tensile testing on small dumb-bell samples taken with an H2-shaped punch in the films before their thermal exposure. These samples were 75 mm long, 12.5 mm wide at both extremities, and about 0.5 mm thick. The useful rectangular part between the two sample heads (i.e., where failure occurs in tension) was 20 mm long, 4 mm wide, and about 0.5 mm thick. These samples were progressively loaded in tension using an Instron 5500K8810/4505H2190 machine (Instron, High Wycombe, UK) with a constant crosshead speed of 50 mm·min^−^^1^ at 23 °C under 50% RH, until their breaking point, according to standards ISO 527-1:2012 and ISO 527-2:2012 [48,49].

For each material and each radio-thermal exposure condition under study, the elongation at break $\varepsilon_{R}$ was plotted as a function of exposure time, and the corresponding lifetime $t_{F}$ was graphically determined using the conventional end-of-life criterion $\varepsilon_{F}$ for electrical cable insulation in the nuclear industry, i.e., $t=t_{F}$ when $\varepsilon_{R}=\varepsilon_{F}=50\%$.

## 3. Results and Discussion

### 3.1. Characterization of the Degradation of the Si-XLPE Matrix

**Figure 3:**
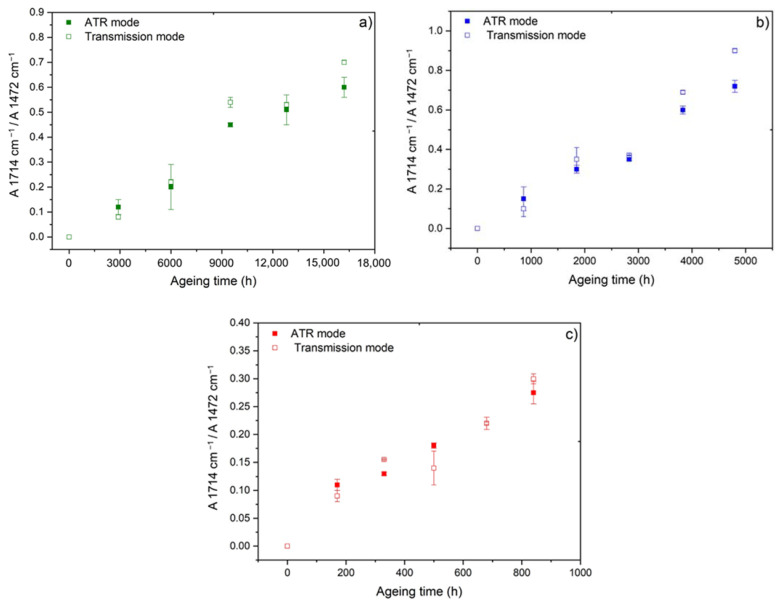
Changes in the carboxylic acid index measured with two different IR methods (i.e., in ATR and transmission modes) for the unfilled Si-XLPE matrix during its radio-thermal exposure in air under 8.5 Gy·h^−1^ at 47 °C (**a**), under 77.8 Gy·h^−1^ at 47 °C (**b**), and under 400 Gy·h^−1^ at 21 °C (**c**).

Figure 3 shows the changes in the carboxylic acid index (i.e., the ratio between the absorbances at 1714 cm^−^^1^ and 1472 cm^−^^1^) during the radio-thermal ageing of the unfilled Si-XLPE matrix in air, for the three dose rates under investigation, at low temperatures close to ambient. To demonstrate the reliability of this oxidation indicator regardless of the IR method under consideration, the values obtained in ATR mode in this study are compared to the values obtained in transmission mode in a previous publication [11]. A satisfactory agreement between both IR methods can clearly be observed, i.e., between an exclusively surface measurement with the ATR mode and an entire volume measurement with the transmission mode. Not only does this satisfactory agreement allow the use of the ATR mode for monitoring the radio-thermal oxidation kinetics in this study to be validated, it also allows the claim that the kinetics is not controlled by the oxygen diffusion, i.e., that films of about 500 µm thick are homogenously oxidized throughout their thickness.

**Figure 4:**
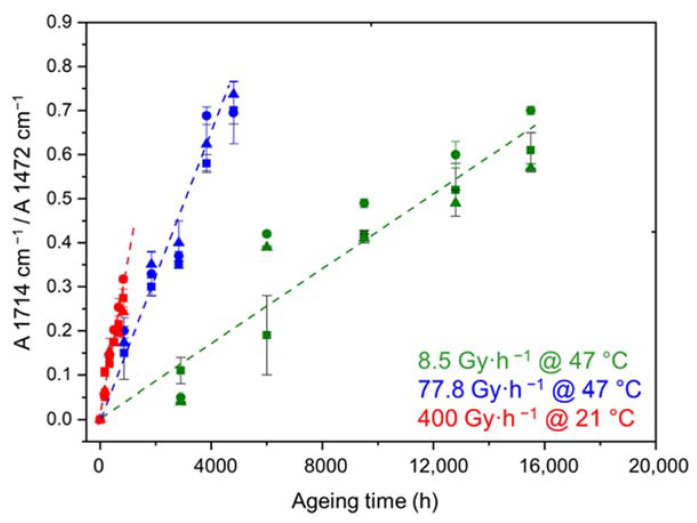
Changes in the carboxylic acid index of the Si-XLPE matrix filled with 0 phr (□), 25 phr (Δ), and 50 phr (○) of ATH, during its radio-thermal exposure in air under 8.5 Gy·h^−1^ at 47 °C (green), under 77.8 Gy·h^−1^ at 47 °C (blue), and under 400 Gy·h^−1^ at 21 °C (red).

Figure 4 shows the changes in the carboxylic acid index during the radio-thermal ageing of the three materials under the three exposure conditions under study. It can clearly be observed that ATH fillers have no influence on the radio-thermal oxidation kinetics of the Si-XLPE matrix. In addition, as already reported for the unfilled Si-XLPE matrix [11], oxidation starts from the early periods of exposure (absence of an induction period) with a maximum rate increasing with the dose rate, which means that oxidation is mainly initiated by the radiolysis of the Si-XLPE matrix.

**Figure 5:**
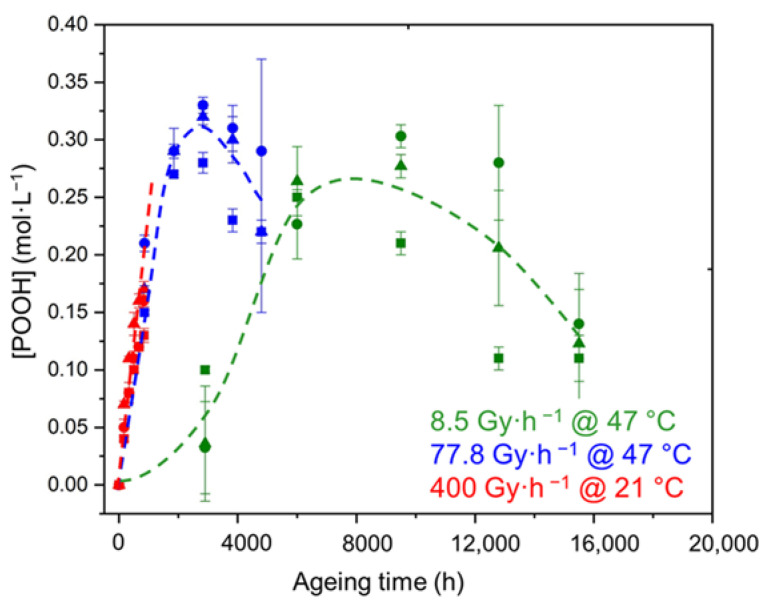
Changes in the POOH concentration of the Si-XLPE matrix filled with 0 phr (□), 25 phr (Δ), and 50 phr (○) of ATH, during its radio-thermal exposure in air under 8.5 Gy·h^−1^ at 47 °C (green), under 77.8 Gy·h^−1^ at 47 °C (blue), and under 400 Gy·h^−1^ at 21 °C (red). Dashed lines are guides for the eye.

The same conclusions can be drawn for the changes in the POOH concentration in the early periods of exposure (see Figure 5). However, it can also be observed that, after a certain duration corresponding to a critical value of the POOH concentration determined in a previous publication [11], the build-up of POOH suddenly slows down, so that the concentration reaches a maximum value and then starts to decrease. This behavior can simply be explained by the fact that the formation of POOH (through the propagation of oxidation) competes with its thermal decomposition. Indeed, as the rate of the thermal decomposition of POOH is proportional to [POOH]^2^ this second initiation reaction can only significantly impact the oxidation kinetics when a sufficient concentration of POOH is formed in the Si-XLPE matrix. Based on this observation, a new kinetic model was recently developed for predicting the radio-thermal oxidation kinetics of the unfilled Si-XLPE matrix [11]. Its main characteristics are briefly recalled in Appendix A.

**Figure 6:**
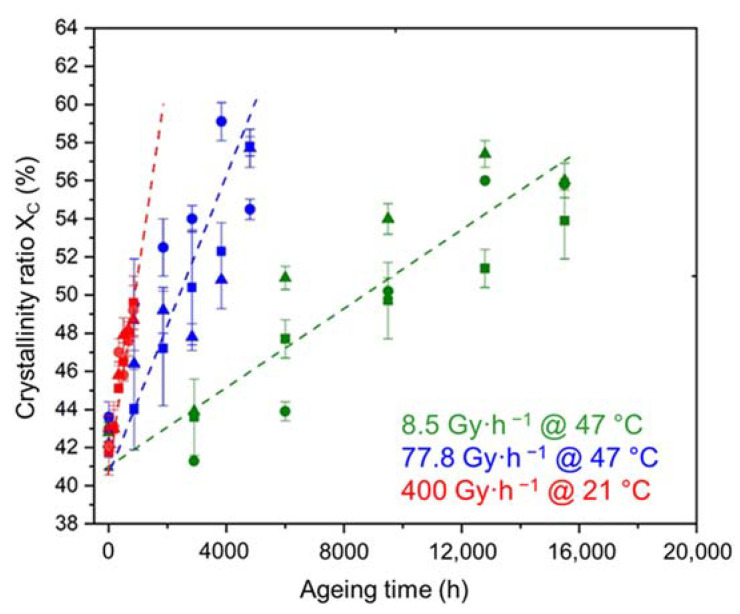
Changes in the mass fraction of crystals in the Si-XLPE matrix filled with 0 phr (□), 25 phr (Δ), and 50 phr (○) of ATH, during its radio-thermal exposure in air under 8.5 Gy·h^−1^ at 47 °C (green), under 77.8 Gy·h^−1^ at 47 °C (blue), and under 400 Gy·h^−1^ at 21 °C (red).

As already explained in Section 2.3, radio-thermal oxidation leads to a predominance of chain scissions over cross-linking, which induces chemi-crystallization, increasing the crystallinity ratio without affecting the melting temperature (i.e., the main crystalline lamellae) of the Si-XLPE matrix. As previously seen for the oxidation kinetics (Figure 4), the ATH fillers also have no influence on the chemi-crystallization kinetics of the Si-XLPE matrix (see Figure 6).

**Figure 7:**
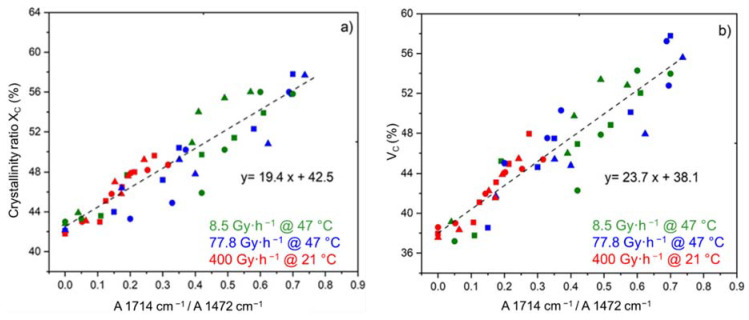
Mass (**a**) and volume fractions of crystals (**b**) versus carboxylic acid index for the Si-XLPE matrix filled with 0 phr (□), 25 phr (Δ), and 50 phr (○) of ATH and aged in air under 8.5 Gy·h^−1^ at 47 °C (green), under 77.8 Gy·h^−1^ at 47 °C (blue), and under 400 Gy·h^−1^ at 21 °C (red).

The two crystallinity ratios $X_{C}$ and $V_{C}$, measured for the three ATH fractions, and the three exposure conditions under study, are plotted as a function of the carboxylic acid index in Figure 7. In both cases, a straight-line relationship was found between the oxidation and chemi-crystallization kinetics, thus confirming that carboxylic acids are, with aldehydes, the main oxidation products from chain scissions in the Si-XLPE matrix. Based on these results, the following two proportionality equations can be proposed:

(17) $$X_{C}=X_{C\;\mathrm{ini}}+19.4\times \frac{A_{1714{\;\mathrm{cm}}^{-1}}}{A_{1472{\;\mathrm{cm}}^{-1}}}$$

(18) $$V_{C}=V_{C\;\mathrm{ini}}+23.7\times \frac{A_{1714{\;\mathrm{cm}}^{-1}}}{A_{1472{\;\mathrm{cm}}^{-1}}}$$

where $X_{C\;\mathrm{ini}}$ and $V_{C\;\mathrm{ini}}$ are the initial values of $X_{C}$ and $V_{C}$, respectively, which are reported in Table 3.

**Figure 8:**
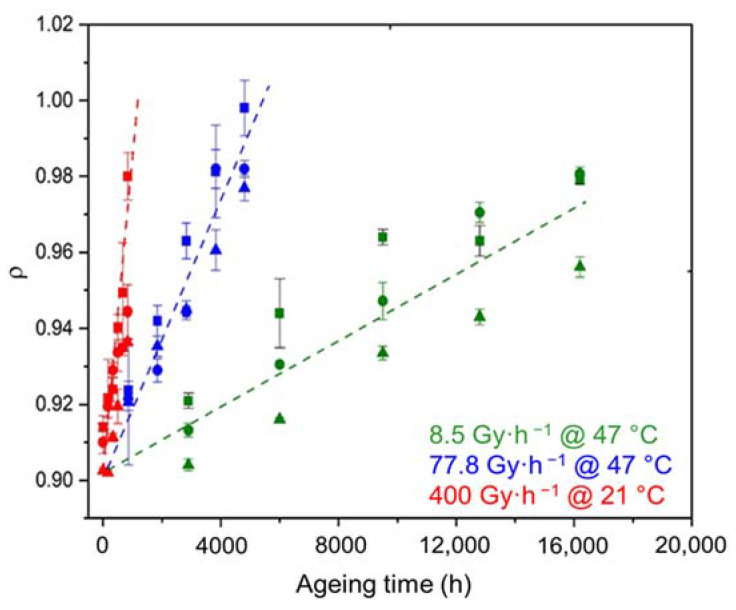
Changes in the density of the Si-XLPE matrix filled with 0 phr (□), 25 phr (Δ), and 50 phr (○) of ATH, during its radio-thermal exposure in air under 8.5 Gy·h^−1^ at 47 °C (green), under 77.8 Gy·h^−1^ at 47 °C (blue), and under 400 Gy·h^−1^ at 21 °C (red).

As shown in Figure 8, chemi-crystallization causes significant changes in the density of the Si-XLPE matrix. As previously seen for both the oxidation (Figure 4) and chemi-crystallization kinetics (Figure 6), the ATH fillers also have no influence on the densification kinetics of the Si-XLPE matrix.

**Figure 9:**
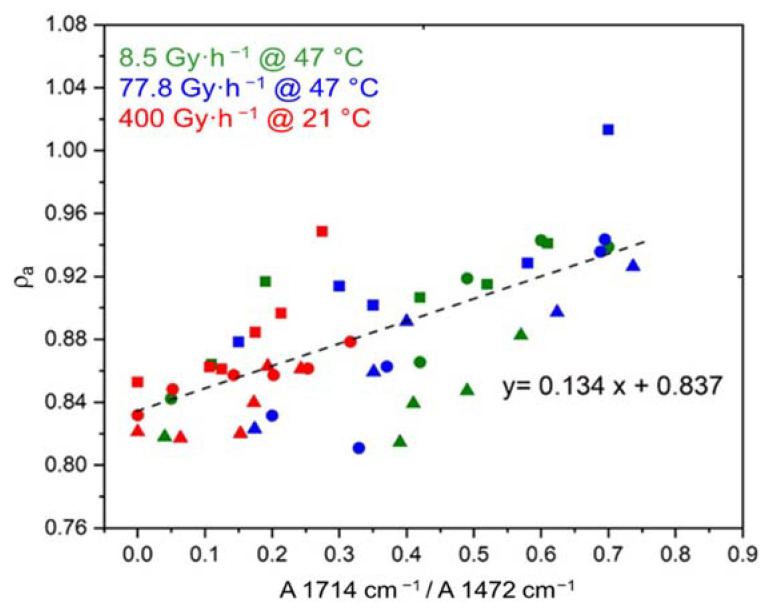
Density of the amorphous phase versus carboxylic acid index for the Si-XLPE matrix filled with 0 phr (□), 25 phr (Δ), and 50 phr (○) of ATH, during its radio-thermal exposure in air under 8.5 Gy·h^−1^ at 47 °C (green), under 77.8 Gy·h^−1^ at 47 °C (blue), and under 400 Gy·h^−1^ at 21 °C (red).

However, chemi-crystallization is not the only phenomenon responsible for the increase in the density of the Si-XLPE matrix during radio-thermal exposure. Indeed, oxygen consumption leads also to a significant increase in the density of its amorphous phase (see Figure 9), so that the total increase in the density of this semi-crystalline polymer must be written as follows [33]:

(19) $$\Delta \rho =\left(1-V_{C\;\mathrm{ini}}\right)\Delta \rho_{a}+\left(\rho_{C}-\rho_{a\;\mathrm{ini}}\right)\Delta V_{C}$$

where $\Delta \rho_{a}$ and $\Delta V_{C}$ designate the increases in the density of the amorphous phase and in the crystallinity ratio, respectively.

**Figure 10:**
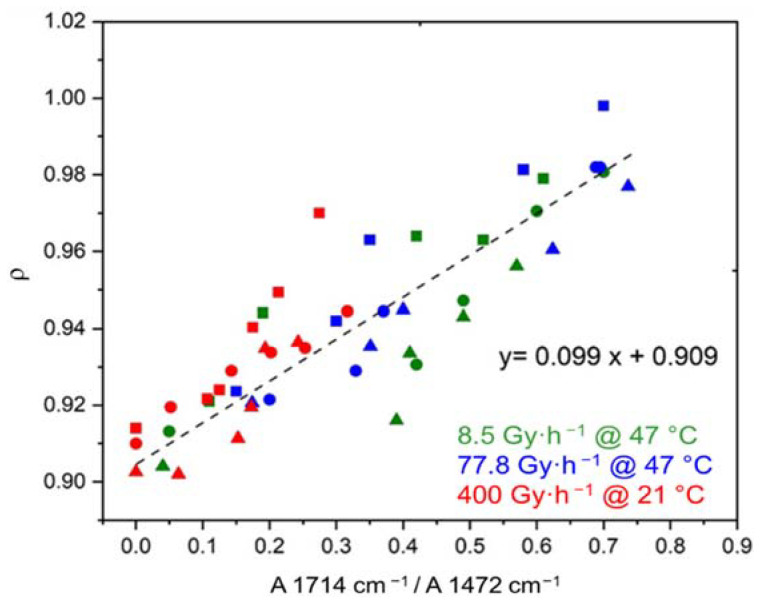
Density versus carboxylic acid index for the Si-XLPE matrix filled with 0 phr (□), 25 phr (Δ), and 50 phr (○) of ATH, during its radio-thermal exposure in air under 8.5 Gy·h^−1^ at 47 °C (green), under 77.8 Gy·h^−1^ at 47 °C (blue), and under 400 Gy·h^−1^ at 21 °C (red).

As previously found for $\Delta V_{C}$ (see Figure 7), $\Delta \rho_{a}$ (Figure 9) and Δρ (Figure 10) are also directly related to the oxidation kinetics through proportionality equations:

(20) $$\rho_{a}=\rho_{a\;\mathrm{ini}}+0.134\times \frac{A_{1714{\;\mathrm{cm}}^{-1}}}{A_{1472{\;\mathrm{cm}}^{-1}}}$$

(21) $$\rho =\rho_{\mathrm{ini}}+0.099\times \frac{A_{1714{\;\mathrm{cm}}^{-1}}}{A_{1472{\;\mathrm{cm}}^{-1}}}$$

where $\rho_{\mathrm{ini}}$ and $\rho_{a\;\mathrm{ini}}$ are the initial values of ρ and $\rho_{a}$, respectively, which are reported in Table 4.

### 3.2. Characterization of the Degradation in the Interfacial Region

**Figure 11:**
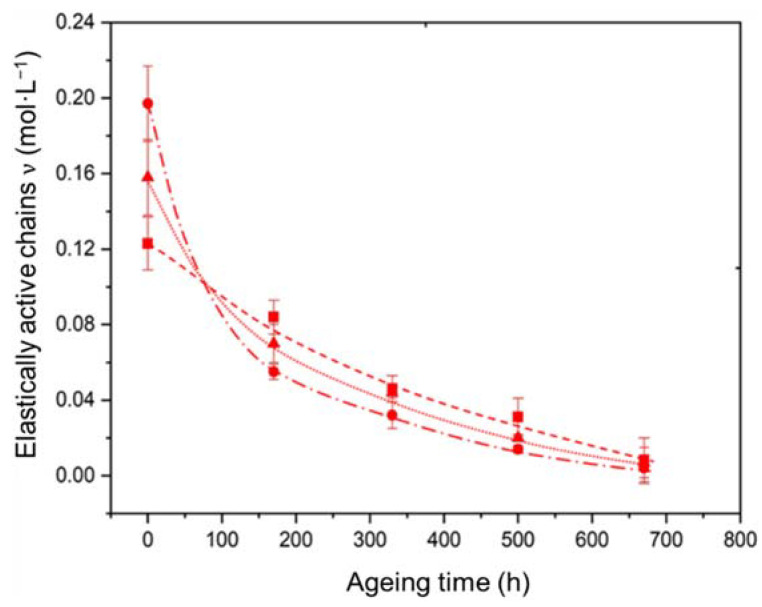
Changes in the concentration of elastically active chains in the Si-XLPE matrix filled with 0 phr (□), 25 phr (Δ), and 50 phr (○) of ATH, during its radio-thermal exposure in air under 400 Gy·h^−1^ at 21 °C.

Figure 11 shows the changes in the concentration of the elastically active chains in the Si-XLPE macromolecular network for the three materials under study during their radio-thermal ageing in air under the highest dose rate (400 Gy·h^−1^). Two major differences are clearly highlighted.

On the one hand, the initial concentration of elastically active chains is clearly an increasing function of the ATH fraction, which is assumed to result from a higher cross-linking density of the Si-XLPE matrix located in the immediate vicinity of the ATH fillers. Indeed, although the ATH fillers do not contain any chemical coupling agent on their surface, Al–OH hydrates can react with specific chemical functions of the starting linear polymer (i.e., Si-g-LDPE) during its immersion in water at 65 °C, for instance with silanols Si-OH, which are formed by hydrolysis of the trimethoxy silane side groups. These additional Al-O-Si covalent bonds are expected to significantly increase the cross-linking density inside the interfacial region.

On the other hand, chain scissions progressively destroy the elastically active chains during radio-thermal exposure, thus creating dangling chains in the Si-XLPE macromolecular network. In the early periods of exposure, the rate of chain scissions is clearly an increasing function of the ATH fraction, because degradation mainly involves the interfacial bonds. In fact, it is suspected that these covalent bonds are poorly resistant to γ-irradiation. Once all these bonds have been rapidly decomposed, typically after less than 100 h of exposure in air under 400 Gy·h^−1^, the rate of chain scissions seems to become independent of the ATH fraction, and thus almost the same values of concentration of elastically active chains are observed for the three materials under study. Thenceforth, the degradation kinetics is almost the same in all the matrix regions (i.e., both inside and outside the interfacial region) of the materials.

**Figure 12:**
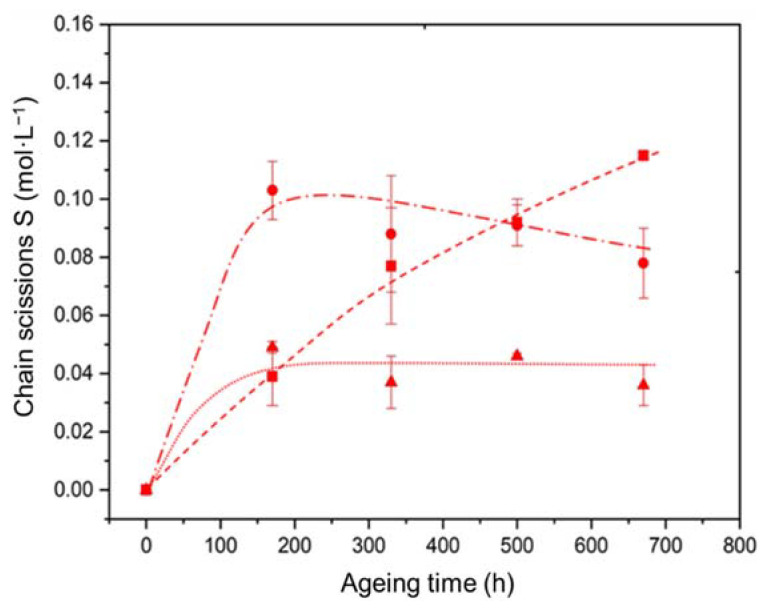
Comparison of the changes in the concentration of chain scissions outside (□) and inside the interfacial region for the two composite materials with 25 phr (Δ) and 50 phr (○) of ATH, during their radio-thermal exposure in air under 400 Gy·h^−1^ at 21 °C.

The corresponding changes in the concentration of chain scissions were calculated using Equation (12) for the three materials under study. Figure 12 compares the changes in the concentration of chain scissions outside ($S_{\mathrm{unf}}$) and inside ($S_{\mathrm{int}}$) the interfacial region for the two composite materials under study. The interfacial changes were simply determined by subtracting the concentrations of chain scissions of the ATH filled and unfilled Si-XLPE matrices, considering that the Si-XLPE matrix located outside the interfacial region exhibits exactly the same behavior as the unfilled Si-XLPE matrix:

(22) $$S_{\mathrm{int}}=S_{\mathrm{fill}}-S_{\mathrm{unf}}$$

It can be observed that the degradation is much higher inside than outside the interfacial region during the first 150 h and 500 h of exposure in air under 400 Gy·h^−1^ for the Si-XLPE matrix filled with 25 phr and 50 phr of ATH, respectively.

During this first period, the changes in mechanical properties that are particularly sensitive to damage, such as fracture properties, are expected to be driven by the degradation of the interfacial degradation. Afterwards, their changes should be further driven by the degradation of all the matrix regions of the materials.

### 3.3. Consequences for Mechanical Properties

**Figure 13:**
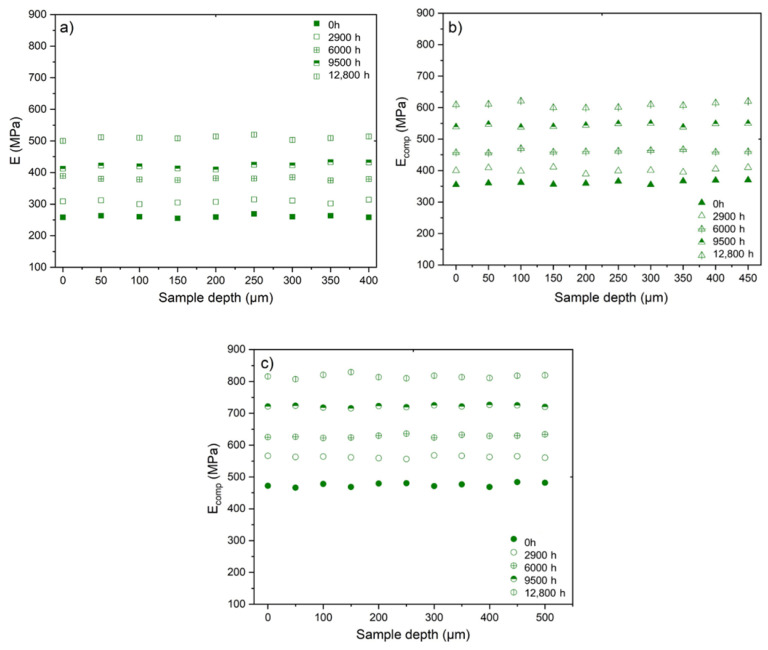
Changes in the profiles of elastic modulus in the film thickness for the unfilled Si-XLPE matrix (**a**) and the two composite materials with 25 phr (**b**) and 50 phr (**c**) of ATH, during their radio-thermal exposure in air under 8.5 Gy·h^−1^ at 47 °C.

Figure 13 shows the changes in the profile of the elastic modulus in the film thickness determined by micro-indention for the three materials under study during their radio-thermal ageing in air under the lowest dose rate (8.5 Gy·h^−1^). It can be observed that these profiles are completely flat, which indicates that the films are homogenously oxidized throughout their thickness. The same conclusions can be drawn for the two other dose rates (i.e., 77.8 Gy·h^−1^ and 400 Gy·h^−1^) under investigation, which justifies once again the choice of the ATR mode to monitor the radio-thermal oxidation kinetics of the Si-XLPE matrix via FTIR spectroscopy in this study.

**Figure 14:**
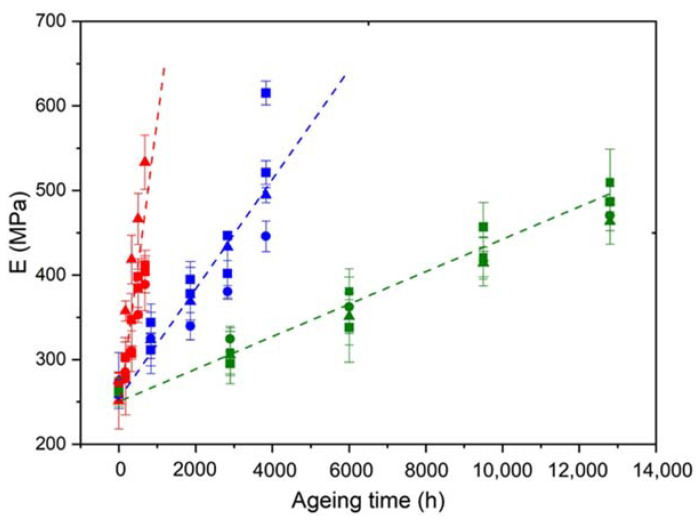
Changes in the elastic modulus of the Si-XLPE matrix filled with 0 phr (□), 25 phr (Δ), and 50 phr (○) of ATH, during its radio-thermal exposure in air under 8.5 Gy·h^−1^ at 47 °C (green), under 77.8 Gy·h^−1^ at 47 °C (blue), and under 400 Gy·h^−1^ at 21 °C (red).

**Figure 15:**
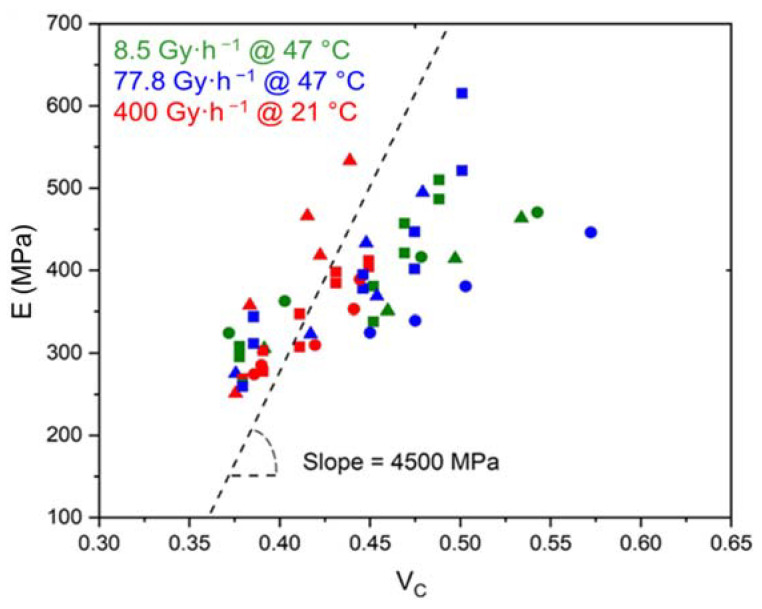
Elastic modulus versus volume fraction of crystals for the Si-XLPE matrix filled with 0 phr (□), 25 phr (Δ), and 50 phr (○) of ATH, during its radio-thermal exposure in air under 8.5 Gy·h^−1^ at 47 °C (green), under 77.8 Gy·h^−1^ at 47 °C (blue), and under 400 Gy·h^−1^ at 21 °C (red).

Figure 14 shows that the elastic modulus of the Si-XLPE matrix linearly increases with the time of exposure for the three materials under the three exposure conditions studied. In Figure 15, despite a fairly large experimental scattering, it can clearly be observed that this linear increase is the direct consequence of the changes in the crystalline morphology of the Si-XLPE matrix, as long as the crystallinity ratio $V_{C}$ does not typically exceed 47%. Indeed, in a first approximation, it can be written:

(23) $$E=\left(1-V_{C}\right)E_{a}+V_{C}E_{C}\;$$

where $E_{a}$ and $E_{C}$ are the Young’s moduli of the amorphous and crystalline phases of the Si-XLPE matrix, respectively. According to the literature, typical values for PE are $E_{a}=$ 4.5 MPa and $E_{C}=$ 4500 MPa [50].

As $E_{C}/E_{a}\approx 1000$, Equation (23) can be simplified as follows:

(24) $$E\approx V_{C}E_{C}\;$$

so that the increase in the Young’s modulus of the Si-XLPE matrix can be written as follows:

(25) $$\Delta E=E_{C}\Delta V_{C}$$

where $\Delta V_{C}$ designates the increase in the crystallinity ratio.

In Figure 15, it can be observed that, below 47% crystallinity, the slope of the curve is effectively equal to $E_{C}=$ 4500 MPa, which allows the validation of Equations (24) and (25).

In contrast, above 47% crystallinity, it would seem that the elastic modulus of the Si-XLPE matrix rapidly tends towards a maximum value independent of the crystallinity ratio. At such high conversion ratios of the oxidation reaction, it is highly probable that the micro-indentation measurements are significantly influenced by the extreme brittleness of the samples. Indeed, it may be suspected that microcracks propagate during the tip penetration into the material. These microcracks should significantly lower the applied load and thus the indentation modulus. For this reason, values of the elastic modulus measured above 47% crystallinity should be regarded with great caution. These values will not be considered for the kinetic modeling.

As already seen for the chemi-crystallization kinetics (Figure 6), the ATH fillers also have no influence on the stiffening kinetics of the Si-XLPE matrix.

**Figure 16:**
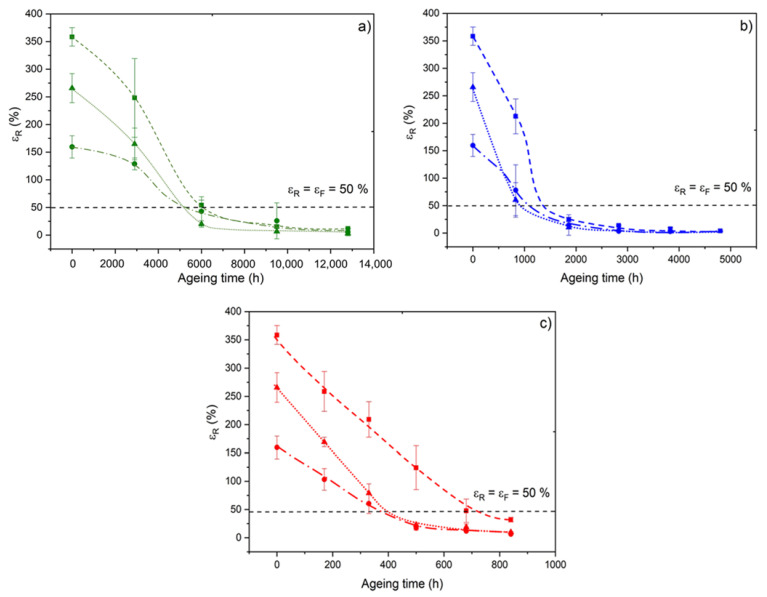
Changes in the elongation at break of the unfilled Si-XLPE matrix (□) and the two composite materials with 25 phr (Δ) and 50 phr (○) of ATH, during their radio-thermal exposure in air under 8.5 Gy·h^−1^ at 47 °C (**a**), under 77.8 Gy·h^−1^ at 47 °C (**b**), and under 400 Gy·h^−1^ at 21 °C (**c**). The horizontal dotted line corresponds to the end-of-life criterion.

Finally, Figure 16 shows the changes in the elongation at break during the radio-thermal ageing of the three materials under the three exposure conditions under study. Two major behavioral trends can be highlighted.

On the one hand, the initial value of the elongation at break is a decreasing function of the ATH fraction. This behavior can be explained by the presence of strong adhesion (involving covalent bonds) between the ATH fillers and the Si-XLPE matrix, which reduces the molecular mobility of the Si-XLPE matrix in the interfacial region.

On the other hand, the elongation at break decreases catastrophically from the early periods of exposure, whatever the ageing conditions under study, due to the rapid destruction of the interfacial bonds and the elastically active chains of the Si-XLPE macromolecular network. The conventional end-of-life criterion for determining the lifetime of polymer insulation in the nuclear industry (i.e., $t=t_{F}$ when $\varepsilon_{F}=\varepsilon_{R}=50\%$) is represented by a horizontal dotted line in Figure 16. The corresponding lifetime values are reported in Table 5. It was found that $t_{F}$ significantly decreases in the presence of ATH fillers. This decrease is even more pronounced when the dose rate is higher. In particular, $t_{F}$ is reduced by half for the two composite materials compared to the unfilled Si-XLPE matrix under the highest dose rate (i.e., 400 Gy·h^−1^). This behavior can be explained by the poor resistance of interfacial bonds to γ-irradiation.

In conclusion, although the interfacial degradation seems to have no impact on the elastic properties (see Figure 15), in contrast it significantly influences the fracture properties (Figure 16 and Table 5).

### 3.4. Extension of the Kinetic Model to Composite Materials

In previous sections, by using different characterization methods, it was shown that the ATH fillers have no influence on the radio-thermal oxidation kinetics of the Si-XLPE matrix. In other words, the role of the ATH fillers is only to reduce the concentration of oxidation products proportionally with the volume fraction ($1-V_{\mathrm{ATH}}$) of the Si-XLPE matrix in the composite material. This is a purely diluting effect. For this reason, the kinetic model previously established for the unfilled Si-XLPE matrix (see Appendix A) was simply extended to composite materials by multiplying the concentrations of the different oxidation products by ($1-V_{\mathrm{ATH}}$). As an example, for hydroperoxides and carbonyls, this can be written:

(26) $${\left[\mathrm{POOH}\right]}_{\mathrm{comp}}=\left(1-V_{\mathrm{ATH}}\right)\left[\mathrm{POOH}\right]$$

(27) $${\left[P=O\right]}_{\mathrm{comp}}=\left(1-V_{\mathrm{ATH}}\right)\left[P=O\right]$$

where ${\left[\mathrm{POOH}\right]}_{\mathrm{comp}}$, ${\left[P=O\right]}_{\mathrm{comp}}$, [POOH], and [P=O] are the concentrations of hydroperoxides and carbonyls in the composite materials and in the unfilled Si-XLPE matrix, respectively. The equations for [POOH] and [P=O] are recalled in Appendix A.

In contrast, the density of the composite materials was determined using the classical mixture law:

(28) $$\rho_{\mathrm{comp}}=\left(1-V_{\mathrm{ATH}}\right)\rho +V_{\mathrm{ATH}}\rho_{\mathrm{ATH}}$$

The equation for ρ is recalled in Appendix A.

**Figure 17:**
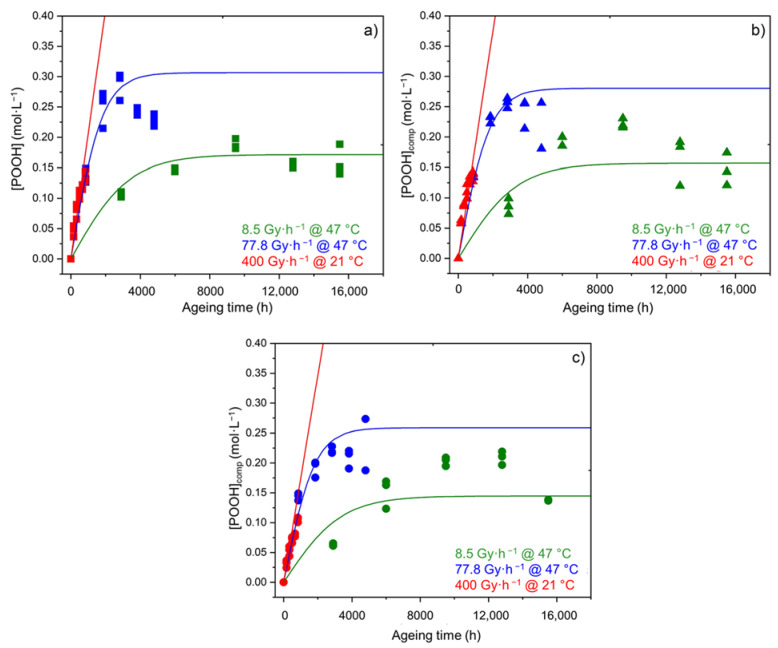
Kinetic modeling of the changes in the hydroperoxide concentration in the unfilled Si-XLPE matrix (**a**) and the two composite materials with 25 phr (**b**) and 50 phr (**c**) of ATH, during their radio-thermal exposure in air under 8.5 Gy·h^−1^ at 47 °C (green), under 77.8 Gy·h^−1^ at 47 °C (blue), and under 400 Gy·h^−1^ at 21 °C (red).

**Figure 18:**
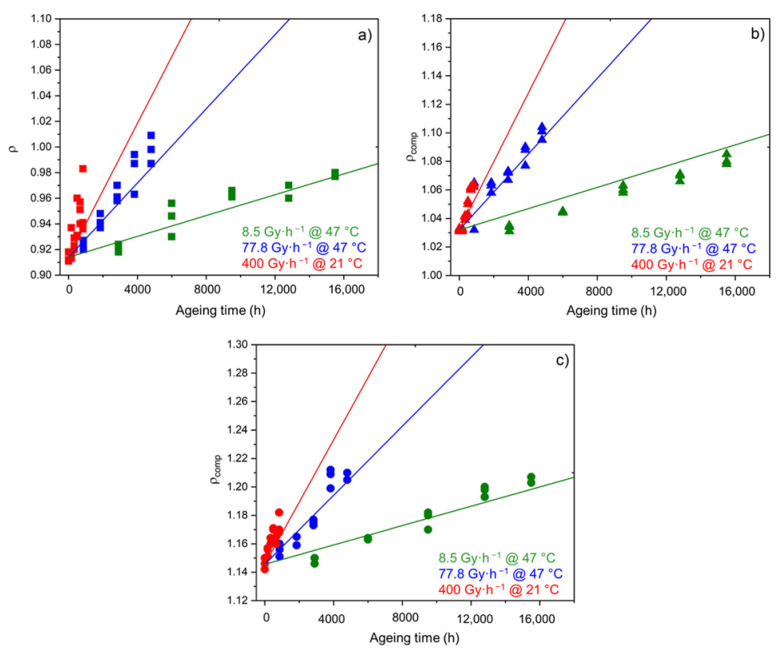
Kinetic modeling of the changes in the hydroperoxide concentration in the unfilled Si-XLPE matrix (**a**) and the two composite materials with 25 phr (**b**) and 50 phr (**c**) of ATH, during their radio-thermal exposure in air under 8.5 Gy·h^−1^ at 47 °C (green), under 77.8 Gy·h^−1^ at 47 °C (blue), and under 400 Gy·h^−1^ at 21 °C (red).

The kinetic modeling of the changes in the hydroperoxide concentration and in the density of the three materials under the three exposure conditions under study is reported in Figure 17 and Figure 18, respectively. In both cases, a satisfactory agreement is obtained between theory and experiment, which allows the reliability of Equations (26) and (28) to be checked.

In Section 3.3, it was also shown that at low conversion ratios of the oxidation reaction, the elastic properties of the composite materials are driven by the crystallinity of the Si-XLPE matrix (see Equation (25)). Introducing Equation (25) into Equation (16) allows the following equation to be proposed for calculating the Young’s modulus of the composite materials:

(29) $$E_{\mathrm{comp}}=\left(1+2.5\times V_{\mathrm{ATH}}+14.1\times V_{\mathrm{ATH}}^{2}\right)\left(E_{\mathrm{ini}}+E_{C}\Delta V_{C}\right)$$

where $E_{\mathrm{ini}}$ is the initial value of the Young’s modulus of the unfilled Si-XLPE matrix. According to the micro-indentation tests, $E_{\mathrm{ini}}=260\pm 15$ MPa for the three materials under study.

The equation for $\Delta V_{C}$ is recalled in Appendix A.

**Figure 19:**
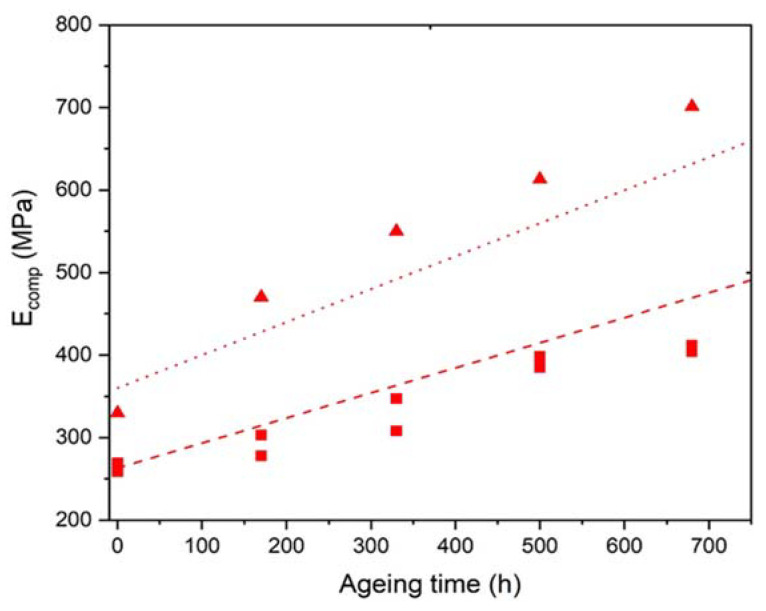
Kinetic modeling of the changes in the elastic modulus of the unfilled Si-XLPE matrix (□) and the composite material with 25 phr of ATH (Δ), during their radio-thermal exposure in air under 400 Gy·h^−1^ at 21 °C.

As also seen in Section 3.3, above 47% crystallinity it is suspected that the micro-indentation results are significantly influenced by the extreme brittleness of the samples. For this reason, the kinetic modeling was limited to the changes in the elastic modulus below this critical crystallinity value. As an example, this is the case for the changes measured for the unfilled Si-XLPE matrix and the composite material with 25 phr ATH in air under the highest dose rate (400 Gy·h^−1^). Figure 19 shows that a satisfactory agreement is obtained between theory and experiment in both cases, thus also confirming the reliability of Equation (29).

Finally, it was shown that the fracture properties are also significantly impacted by the degradation of the interfacial region. For this reason, a global end-of-life criterion, summing all damages of the Si-XLPE matrix, i.e., those occurring both inside and outside the interfacial region, was proposed for composite materials:

(30) $$S_{\mathrm{comp}}=\left(1-V_{\mathrm{ATH}}\right)\left(S+S_{\mathrm{int}}\right)$$

The equation for S is recalled in Appendix A.

In agreement with the experimental results in the two last sections (Section 3.2 and Section 3.3), it was assumed that the radiolytic decomposition of the interfacial bonds obeys an apparent first-order kinetics whose rate constant is proportional to dose rate:

(31) $$\frac{d\left[\mathrm{Al}-O-\mathrm{Si}\right]}{\mathrm{dt}}=-k_{7}\left[\mathrm{Al}-O-\mathrm{Si}\right]$$

with

(32) $$k_{7}=\phi \times I\;$$

where ϕ is a proportionality coefficient and I is the dose rate expressed in Gy·s^−1^. The value of ϕ was set at $10^{-4}$ so that all the interfacial bonds were decomposed after only 100 h of exposure under the highest dose rate, as experimentally observed in Figure 11 and Figure 12.

Equation (31) can be rewritten as:

(33) $$\frac{d\left[\mathrm{Al}-O-\mathrm{Si}\right]}{\left[\mathrm{Al}-O-\mathrm{Si}\right]}=-k_{7}\mathrm{dt}\;$$

i.e.,

(34) $$\left[\mathrm{Al}-O-\mathrm{Si}\right]={\left[\mathrm{Al}-O-\mathrm{Si}\right]}_{\mathrm{ini}}\mathrm{Exp}\left(-k_{7}t\right)$$

where ${\left[\mathrm{Al}-O-\mathrm{Si}\right]}_{\mathrm{ini}}$ is the initial concentration of interfacial bonds.

The concentration of chain scissions is given by:

(35) $$\frac{{\mathrm{dS}}_{\mathrm{int}}}{\mathrm{dt}}=k_{7}{\left[\mathrm{Al}-O-\mathrm{Si}\right]}_{\mathrm{ini}}\mathrm{Exp}\left(-k_{7}t\right)$$

i.e.,

(36) $$S_{\mathrm{int}}={\left[\mathrm{Al}-O-\mathrm{Si}\right]}_{\mathrm{ini}}\left(1-\mathrm{Exp}\left(-k_{7}t\right)\right)$$

According to Figure 12, ${\left[\mathrm{Al}-O-\mathrm{Si}\right]}_{\mathrm{ini}}$ is an increasing function of $V_{\mathrm{ATH}}$. From the maximum values of $S_{\mathrm{int}}$ obtained for the two composite materials, it can be deduced that about 57% of the ATH fillers establish a covalent bond with the Si-XLPE matrix:

(37) $${\left[\mathrm{Al}-O-\mathrm{Si}\right]}_{\mathrm{ini}}=0.57\times V_{\mathrm{ATH}}$$

**Figure 20:**
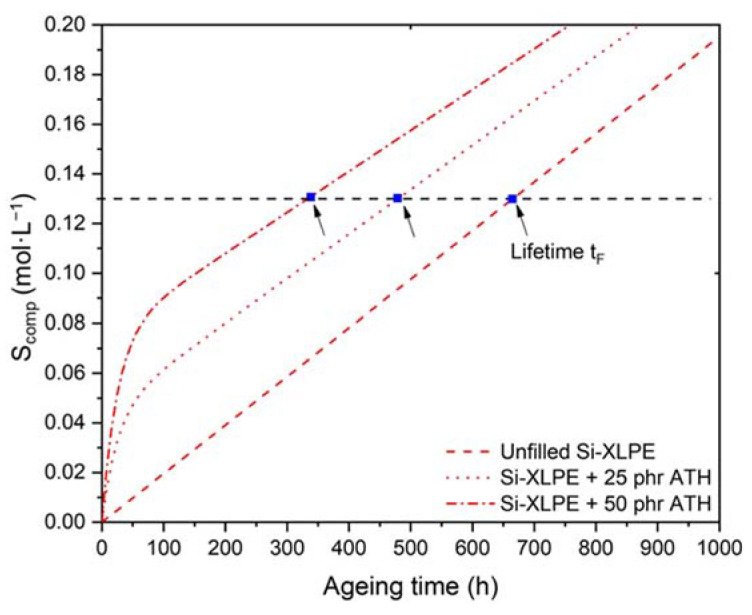
Prediction of the lifetime for the unfilled Si-XLPE matrix and the composite materials with 25 phr and 50 phr of ATH, from the changes in their concentration of chain scissions during radio-thermal exposure in air under 400 Gy·h^−1^ at 21 °C. The horizontal dotted line corresponds to the end-of-life criterion.

It should be recalled that the lifetime of the unfilled Si-XLPE matrix can be predicted using a critical concentration of hydroperoxides ${\left[\mathrm{POOH}\right]}_{F}\approx \left(1.6\pm 0.2\right)\times 10^{-1}$ mol·L^−1^ as a structural end-of-life criterion [11], which corresponds to a critical concentration of chain scissions $S_{F}\approx \left(1.3\pm 0.2\right)\times 10^{-1}$ mol·L^−1^. Assuming that this remains valid regardless of the ATH fraction, this last criterion was also used to determine the lifetime of the two composite materials, as shown in Figure 20, where the changes in the concentration of chain scissions predicted using Equation (21) are plotted for the highest dose rate (400 Gy·h^−1^). The structural end-of-life criterion for determining the lifetime (i.e., $t=t_{F}$ when $S_{\mathrm{comp}}=S_{F}=\left(1.3\pm 0.2\right)\times 10^{-1}$ mol·L^−1^) is represented by a horizontal dotted line. The lifetime values determined for all exposure conditions under investigation are reported in Table 6.

A satisfactory agreement can be observed between the measured and predicted lifetime values reported in Table 5 and Table 6, respectively, showing the high predictive quality of the kinetic modeling approach developed in this study and validating the extension to composite materials.

## 4. Conclusions

This study aimed at highlighting the possible effect of ATH fillers on the radio-thermal ageing of a Si-XLPE matrix using both a multi-scale and a multi-technique approach. The same changes at the molecular and morphological scales were observed regardless of the ATH fraction in the Si-XLPE matrix. In addition, it was clearly shown that the ATH fillers have no influence on the oxidation and crystallization kinetics, thus leading to similar densification kinetics of the Si-XLPE matrix. Finally, it was found that the changes in the elastic properties of the three materials under study are mainly driven by the crystallinity of the Si-XLPE matrix, whereas the changes in their fracture properties are also significantly impacted by the degradation of the ATH/Si-XLPE interface, as proven by decrease in the lifetime $t_{F}$ when increasing the ATH fraction in the Si-XLPE matrix.

Based on all these experimental observations, the kinetic model previously developed for the unfilled Si-XLPE was extended to composite materials by taking into account both the diluting effect of ATH fillers (i.e., the ATH content) and the interfacial degradation. The reliability of this new kinetic model was successfully checked by comparing its predictions with the changes in several physico-chemical and mechanical properties (hydroperoxide concentration, density, Young’s modulus, etc.) for the three materials under the three exposure conditions under study. In addition, the lifetimes of the three materials were successfully predicted from the changes in the concentration of chain scissions using a global end-of-life criterion, taking into account the matrix degradation both inside and outside the interfacial region. Experiments and kinetic modeling showed the poor resistance of the interfacial bonds to γ-irradiation, in addition to the well-known high sensitivity of the Si-XLPE matrix to oxidation [11,33]. In contrast to previous claims in the literature [21,22,23], the radiolytic decomposition of the interfacial bonds did not lead to the formation of additional radical species since no acceleration of the oxidation kinetics was detected.

An interesting perspective would be to generalize this kinetic modeling approach to other types of composite materials.

## Figures and Tables

**Table 1:** Physico-chemical properties of the as-received materials and their Si-XLPE matrix, where ρ_comp_, X_ATH_, and V_ATH_ are the density, mass, and volume fraction, respectively, of ATH in the composite materials. In addition, ρ, X_C_, and F_g_ are the density, crystallinity ratio, and gel content, respectively, of the Si-XLPE matrix.

	ρ_comp_	X_ATH_ (%)	V_ATH_ (%)	ρ	X_C_ (%)	F_g_ (%)
**Unfilled Si-XLPE**	-	0	0	0.914	42.1	71.1
**Si-XLPE + 25 phr ATH**	1.032	20.9	8.5	0.903	42.8	69.6
**Si-XLPE + 50 phr ATH**	1.146	33.2	15.6	0.910	43.0	67.6

**Table 2:** Radio-thermal ageing conditions.

Dose Rate (Gy·h^−1^)	Dose Rate (Gy·s^−1^)	Temperature (°C)	Withdrawal Times of Samples (h)	Withdrawal Doses of Samples (kGy)
8.5	2.36 × 10^−3^	47	2900–6000–9500–12,800–15,500	25–51–81–109–132
77.8	2.16 × 10^−2^	47	860–1850–2830–3830–4800	67–144–220–298–373
400	1.11 × 10^−1^	21	167–334–501–668–835	68–134–200–267–334

**Table 3:** Main degradation products detected by FTIR spectroscopy during the radio-thermal ageing of the Si-XLPE matrix.

Degradation Products	Wavenumbers (cm^−1^)	References
Conjugated C=C	1590 and 1630	[30]
Isolated C=C	1650	[30]
Carboxylic acids	1714	[11]
Aldehydes	1736	[11]
γ-Lactones or anhydrides	1778	[11]

**Table 4:** Physical properties of the Si-XLPE matrix of the as-received materials, where $\rho_{\mathrm{ini}}$, $X_{C\;\mathrm{ini}}$, $V_{C\;\mathrm{ini}}$, and $\rho_{a\;\mathrm{ini}}$ are its density, the mass and volume fractions of crystals, and the density of its amorphous phase, respectively.

	$\rho_{ini}$	$X_{C\;ini}\;(\%)$	$V_{C\;ini}\;(\%)$	$\rho_{a\;ini}$
**Unfilled Si-XLPE**	0.914	42.1	37.9	0.853
**Si-XLPE + 25 phr ATH**	0.903	42.8	37.6	0.821
**Si-XLPE + 50 phr ATH**	0.910	43.0	38.6	0.832
**Average value**	0.909 ± 0.006	42.5 ± 0.5	38.1 ± 0.5	0.837 ± 0.016

**Table 5:** Lifetime values (expressed in hours) graphically determined for the three materials under the three exposure conditions under study, as shown in Figure 16.

	8.5 Gy·h^−1^ ≅ 47 °C	77.8 Gy·h^−1^ ≅ 47 °C	400 Gy·h^−1^ ≅ 21 °C
**Unfilled Si-XLPE**	6240	1200	720
**Si-XLPE + 25 phr ATH**	5300	1000	400
**Si-XLPE + 50 phr ATH**	5300	1100	370

**Table 6:** Lifetime values (expressed in hours) graphically determined for the three materials under the three exposure conditions under study, as shown in Figure 20.

	8.5 Gy·h^−1^ ≅ 47 °C	77.8 Gy·h^−1^ ≅ 47 °C	400 Gy·h^−1^ ≅ 21 °C
**Unfilled Si-XLPE**	6500	1250	670
**Si-XLPE + 25 phr ATH**	5300	1250	480
**Si-XLPE + 50 phr ATH**	5300	1000	340

## Data Availability

The data presented in this study are available on request from the corresponding author.

## Appendix A. Quick Summary of the Kinetic Modeling of the Radio-Thermal Ageing of the Unfilled Si-XLPE Matrix

### Appendix A.1. Mechanistic Scheme

The kinetic model for describing radio-thermal oxidation of the unfilled Si-XLPE matrix in the domain of practical interest for nuclear power plant operators (typically for dose rates I ranging between 1.6 × 10^−1^ Gy·s^−1^ and 5.0 × 10^−1^ Gy·s^−1^) was established in two previous publications [11,33]. It is derived from a mechanistic scheme in which oxidation is initiated by both the polymer radiolysis (1R) and the thermal decomposition of POOH in bimolecular mode (1T):

Initiation:

(1R) PH + hν → P^•^ + ½H_2_   ($r_{i}=10^{-7}G_{i}I$)
(1T) 2POOH → P^•^ + PO_2_^•^   ($k_{1}$)

Propagation:

(2) P^•^ + O_2_ → PO_2_^•^  ($k_{2}$)
(3) PO_2_^•^ + PH → POOH + P^•^ ($k_{3}$)

Terminations:

(4) P^•^ + P^•^ → Inactive products  ($k_{4}$)
(5) P^•^ + PO_2_^•^ → Inactive products   ($k_{5}$)
(6) PO_2_^•^ + PO_2_^•^ → Inactive products + O_2_ ($k_{6}$)

where PH, POOH, P^•^, and PO_2_^•^ designate an oxidation site, a hydroperoxide, and alkyl and peroxy radicals, respectively. In addition, $r_{i}$, $G_{i}$, and $k_{j}$ (with j = 1, … 6) are the radiolytic initiation rate, the radical yield, and the rate constants, respectively.

### Appendix A.2. Kinetic Model

The system of differential equations derived from this mechanistic scheme can be solved analytically using only two simplifying (but realistic) assumptions [11].

Oxidation is mainly initiated by polymer radiolysis throughout the exposure (i.e., $r_{i}\gg 2k_{1}{\left[\mathrm{POOH}\right]}^{2}$), and the thermal decomposition of POOH is an additional (but secondary) source of radicals in the long term.

Radical species reach a steady-state regime from the early periods of the radio-thermal exposure (i.e., d[Rad]/dt=0).

By using only these two assumptions, the following equations were found for predicting the following chemical properties [11]:

The concentration of POOH:

(A1) $$\left[\mathrm{POOH}\right]={\left[\mathrm{POOH}\right]}_{\infty }\frac{1-b\;\mathrm{Exp}\left(-\mathrm{Kt}\right)}{1+b\;\mathrm{Exp}\left(-\mathrm{Kt}\right)}$$

with

(A2) $${\left[\mathrm{POOH}\right]}_{\infty }={\left(\frac{k_{3}\left[\mathrm{PH}\right]}{2k_{1b}}{\left(\frac{r_{i}}{2k_{6}}\right)}^{1/2}\frac{\beta C}{1+\beta C}\right)}^{1/2}$$

(A3) $$K=2{\left(2k_{3}\left[\mathrm{PH}\right]k_{1b}{\left(\frac{r_{i}}{2k_{6}}\right)}^{1/2}\frac{\beta C}{1+\beta C}\right)}^{1/2}$$

and

(A4) $$b=\frac{{\left[\mathrm{POOH}\right]}_{\infty }-{\left[\mathrm{POOH}\right]}_{\mathrm{ini}}}{{\left[\mathrm{POOH}\right]}_{\infty }+{\left[\mathrm{POOH}\right]}_{\mathrm{ini}}}$$

where ${\left[\mathrm{POOH}\right]}_{\mathrm{ini}}$ and ${\left[\mathrm{POOH}\right]}_{\infty }$ are the initial and steady concentrations of hydroperoxides, respectively. For weakly pre-oxidized samples, it is usually observed that ${\left[\mathrm{POOH}\right]}_{\infty }\gg {\left[\mathrm{POOH}\right]}_{\mathrm{ini}}$ [29,51], and therefore it can be considered that b≈1.

The oxygen consumption is:

(A5) $$Q_{O2}=\left[k_{3}\left[\mathrm{PH}\right]{\left(\frac{r_{i}}{2k_{6}}\right)}^{1/2}\frac{\beta C}{1+\beta C}+r_{i}\frac{\beta C}{1+\beta C}\left(1-\frac{\beta C}{2\left(1+\beta C\right)}\right)\right]t$$

The carbonyl concentration is:

(A6) $$\begin{array}{c} \left[P=O\right]=\left[\gamma_{1\mathrm{CO}}\frac{k_{3}\left[\mathrm{PH}\right]}{2}{\left(\frac{r_{i}}{2k_{6}}\right)}^{1/2}\frac{\beta C}{1+\beta C}+\gamma_{6\mathrm{CO}}\frac{r_{i}}{2}{\left(\frac{\beta C}{1+\beta C}\right)}^{2}\right]t\;\\ +2\gamma_{1\mathrm{CO}}\frac{k_{3}\left[\mathrm{PH}\right]}{K}{\left(\frac{r_{i}}{2k_{6}}\right)}^{1/2}\frac{\beta C}{1+\beta C}\left(\frac{1}{1+b\;\mathrm{Exp}\left(-\mathrm{Kt}\right)}-\frac{1}{1+b}\right) \end{array}$$

where $\gamma_{1\mathrm{CO}}$ and $\gamma_{6\mathrm{CO}}$ are the respective formation yields of carbonyls in thermal initiation (1T) and termination (6).

The concentration of chain scissions is:

(A7) $$\begin{array}{c} S=\left[\gamma_{1S}\frac{k_{3}\left[\mathrm{PH}\right]}{2}{\left(\frac{r_{i}}{2k_{6}}\right)}^{1/2}\frac{\beta C}{1+\beta C}+\gamma_{6S}\frac{r_{i}}{2}{\left(\frac{\beta C}{1+\beta C}\right)}^{2}\right]t\;\\ \;\;+2\gamma_{1S}\frac{k_{3}\left[\mathrm{PH}\right]}{K}{\left(\frac{r_{i}}{2k_{6}}\right)}^{1/2}\frac{\beta C}{1+\beta C}\left(\frac{1}{1+b\;\mathrm{Exp}\left(-\mathrm{Kt}\right)}-\frac{1}{1+b}\right) \end{array}$$

where $\gamma_{1S}$ and $\gamma_{6S}$ are the respective yields of chain scissions in thermal initiation (1T) and termination (6).

In Equations (A2), (A3), and (A5)–(A7), C is the oxygen concentration in the Si-XLPE matrix, which is related to the oxygen partial pressure $P_{O2}$ in the exposure environment by the classical Henry’s law:

(A8) $$C=S\times P_{O2}$$

where S is the coefficient of oxygen solubility in the Si-XLPE matrix. Typical values of S reported for low-density polyethylene (LDPE) in the literature are about 1.8 × 10^−8^ mol·L^−1^·Pa^−1^, regardless of the temperature [52]. As an example, in the case of ageing in air under atmospheric pressure with $P_{O2}=0.21\times 10^{5}$ Pa, we obtain $C=3.8\times 10^{-4}$ mol·L^−1^.

In addition, $\beta^{-1}$ corresponds to the critical value of the oxygen concentration $C_{C}$ above which oxygen excess is reached:

(A9) $$\beta =\frac{1}{C_{C}}\approx \frac{2k_{6}k_{2}}{k_{5}\left[k_{3}\left[\mathrm{PH}\right]+{\left(2r_{i}k_{6}\right)}^{1/2}\right]}$$

More recently, it was found that the increase in the density of a semi-crystalline polymer during radio-thermal exposure results from two contributions: oxygen consumption and chemi-crystallization, which led us to propose the following equation for the Si-XLPE matrix [33]:

(A10) $$\Delta \rho =\left(1-V_{C\;\mathrm{ini}}\right)\Delta \rho_{a}+\left(\rho_{C}-\rho_{a\;\mathrm{ini}}\right)\Delta V_{C}$$

where $\Delta \rho_{a}$ and $\Delta V_{C}$ designate the increases in the density of the amorphous phase and the crystallinity ratio, respectively, $\rho_{a\;\mathrm{ini}}$ and $V_{C\;\mathrm{ini}}$ are the initial values of the density of the amorphous phase and the crystalline ratio, respectively, and $\rho_{C}$ is the density of the crystalline phase.

In addition, for the Si-XLPE matrix, it was shown that [33]:

(A11) $$\Delta \rho_{a}=7.03\times 10^{-2}\times Q_{O2}$$

and

(A12) $$\Delta V_{C}=1.96\times 10^{-1}\times Q_{O2}$$

$Q_{O2}$ is expressed in mol·L^−1^.

Introducing Equations (A11) and (A12) into Equation (A10) leads finally to:

(A13) $$\Delta \rho =\left[7.03\times 10^{-2}\left(1-V_{C\;\mathrm{ini}}\right)+1.96\times 10^{-1}\left(\rho_{C}-\rho_{a\;\mathrm{ini}}\right)\right]\times Q_{O2}$$

i.e.,

(A14) $$\rho =\rho_{\mathrm{ini}}+\left[7.03\times 10^{-2}\left(1-V_{C\;\mathrm{ini}}\right)+1.96\times 10^{-1}\left(\rho_{C}-\rho_{a\;\mathrm{ini}}\right)\right]\times Q_{O2}$$

### Appendix A.3. Parameters

The values of the various unknown parameters in Equations (A1)–(A14) (i.e., the radical yield $G_{i}$, the rate constants $k_{j}$ (with j = 1, …, 6), the formation yields of carboxylic acids $\gamma_{1\mathrm{CO}}$ and $\gamma_{6\mathrm{CO}}$, and the yields of chain scissions $\gamma_{1S}$ and $\gamma_{6S}$) that were used for the kinetic modeling of the radio-thermal oxidation of the unfilled Si-XLPE matrix in two recent publications [11,33] are shown in Table A1.

**Table A1:** Values of parameters used for modeling the oxidation kinetics of the unfilled Si-XLPE matrix in the various radio-thermal environments under study [11,33].

T (°C)	21	47	47
**I** **(Gy·h^−1^)**	400	77.8	8.5
$G_{i}$	8	8	8
$k_{1b}$ **(** **L·mol** ^**−1**^ **·s** ^**−1**^ **)**	5.0 × 10^−9^	2.5 × 10^−7^	2.4 × 10^−7^
$k_{2}$ **(L·mol** ^**−1**^ **·s** ^**−1**^ **)**	10^8^	10^8^	10^8^
$k_{3}$ **(L·mol** ^**−1**^ **·s** ^**−1**^ **)**	1.6 × 10^−3^	1.9 × 10^−2^	1.9 × 10^−2^
$k_{4}$ **(L·mol** ^**−1**^ **·s** ^**−1**^ **)**	8.0 × 10^11^	8.0 × 10^11^	8.0 × 10^11^
$k_{5}$ **(L·mol** ^**−1**^ **·s** ^**−1**^ **)**	1.2 × 10^10^	7.0 × 10^10^	9.0 × 10^10^
$k_{6}$ **(L·mol** ^**−1**^ **·s** ^**−1**^ **)**	5.0 × 10^4^	1.0 × 10^6^	2.0 × 10^6^
$\gamma_{1CO}$ **(%)**	90	70	75
$\gamma_{6CO}$ **(%)**	90	70	75
$\gamma_{1S}$ **(%)**	90	52	52
$\gamma_{6S}$ **(%)**	90	52	52
